# Retraction Note: Copanlisib promotes growth inhibition and apoptosis by modulating the AKT/FoxO3a/PUMA axis in colorectal cancer

**DOI:** 10.1038/s41419-025-07679-w

**Published:** 2025-04-29

**Authors:** Ji Yan, Shida Yang, Hong Tian, Yang Zhang, Hongmei Zhao

**Affiliations:** 1 Department of Medicine Laboratory, The 4th People’s Hospital of Shenyang, Shenyang, Liaoning China; 2 Department of Laboratory Medicine, The People’s Hospital of China Medical University (The People’s Hospital of Liaoning Province), Shenyang, Liaoning China; 3 Oncology Department, The 4th People’s Hospital of Shenyang, Shenyang, Liaoning China; 4 Department of Pathology, The 4th People’s Hospital of Shenyang, Shenyang, Liaoning China

Retraction Note to: *Cell Death and Disease* 10.1038/s41419-020-03154-w, published online 02 November 2020

The Editors-in-Chief have retracted this article because of concerns regarding the figures presented in this work. These concerns call into question the article’s overall scientific soundness. An investigation conducted after its publication discovered the following issues:

Panel PDX1, Untreated in Figure 5E appears to overlap, when re-scaled, with Panel Lestaurtinib, WT in Figure 6F in [1];

Panel PDX1, Untreated in Figure 5F appears to overlap, when re-scaled, with Panel Untreated, PUMA-KO in Figure 6C in [2].

The panels in question represent tissues taken from animals subject to different experimental conditions. The Editors-in-Chief therefore no longer have confidence in the integrity of the research presented in this article.

The authors have not replied to correspondence from the Publisher about this retraction.
